# Vasomotor Symptom Trajectories and Risk of Incident Diabetes

**DOI:** 10.1001/jamanetworkopen.2024.43546

**Published:** 2024-10-31

**Authors:** Monique M. Hedderson, Emily F. Liu, Catherine Lee, Samar R. El Khoudary, Ellen B. Gold, Carol A. Derby, Rebecca C. Thurston

**Affiliations:** 1 Division of Research, Kaiser Permanente Northern California, Pleasanton; 2 Department of Epidemiology, University of Pittsburgh Graduate School of Public Health, Pittsburgh, Pennsylvania; 3 Department of Public Health Sciences, School of Medicine, University of California, Davis; 4 Department of Psychiatry, University of Pittsburgh, Pennsylvania; 5 Departments of Neurology and Epidemiology and Population Health, Albert Einstein College of Medicine, Bronx, New York

## Abstract

This cohort study evaluates the association between frequency and trajectories of vasomotor symptoms with incident type 2 diabetes among premenopausal or perimenopausal women in the US.

## Introduction

Vasomotor symptoms (VMS; night sweats and hot flashes) are the hallmark signs of menopause. Growing evidence suggests an association between VMS and the increased cardiometabolic risk experienced in women during and after the menopausal transition (MT).^1^ While most women experience VMS,^2^ the frequency and the temporal patterns vary dramatically across subgroups of women.^3^ Past studies examined the association of VMS at 1 time point with diabetes; thus, it remains unknown whether VMS trajectories across the MT are associated with risk of type 2 diabetes (T2D). We examined the associations of frequency and trajectories of VMS with incident T2D over the MT in the Study of Women’s Health Across the Nation (SWAN).

## Methods

SWAN is a prospective cohort of US women who were premenopausal or early perimenopausal at baseline and assessed at up to 13 approximately annual follow-up visits at 7 clinical sites (eTable).^4^ All protocols were approved by the sites’ institutional review boards, and all study participants provided informed written consent. This study followed the Strengthening the Reporting of Observational Studies in Epidemiology (STROBE) reporting guideline. The analytic sample excluded participants who had diabetes or used exogenous hormones at baseline, did not have any follow-up visits or at least 3 visits before diabetes diagnosis, or were missing VMS information.

At each follow-up visit, participants reported how often they experienced hot flashes and/or night sweats in the past 2 weeks. Women were defined as having diabetes at each visit if they reported use of antidiabetic medication, had 2 consecutive visits with fasting glucose 126 mg/dL (to convert to mmol/L, multiply by .0555) or more while not receiving steroids, or had 2 visits with self-reported diabetes and 1 visit with fasting glucose 126 mg/dL or more (eMethods in Supplement 1).

We tested associations between VMS and VMS trajectories and incident diabetes using discrete-time hazard models^5^ via fitted generalized linear models with a complementary log-log link to generate hazard ratios (HRs) and 95% CIs. VMS were also modeled using a group-based trajectory approach^6^ to identify VMS patterns during follow-up. All models were adjusted for time (study visit) and site; minimally adjusted models included self-reported race and ethnicity (Black, Chinese, Hispanic, Japanese, and White; assessed because of concerns regarding differences in health outcomes across these groups), baseline age and educational attainment, and time-varying body mass index (BMI), physical activity, smoking status, alcohol consumption, and MT stage; fully adjusted models additionally included sex hormones (eMethods in Supplement 1). Analyses were conducted in RStudio version 2023.09.1 (R Project for Statistical Computing). All results were considered statistically significant at a 2-sided *P* ≤ .05. Data were analyzed from January 2021 to June 2024.

## Results

A total of 2761 SWAN participants at baseline were midlife and racially and ethnically diverse (mean [SD] age, 46 [3] years; 737 [27%] Black, 265 [9.6%] Japanese, and 1345 [49%] White) (Table 1). At baseline, 764 (28%) reported VMS 1 to 5 days per 2-week period, 280 (10%) reported VMS 6 or more days per week, and 1716 (62%) reported no VMS; 338 (12.2%) developed diabetes during follow-up (Table 1).

**Table 1.** Baseline Characteristics of Study Sample From Study of Women’s Health Across the Nation Population

Characteristic	Patients, No. (%) (N = 2761)
Age, mean (SD), y	46 (3)
Vasomotor symptom frequency, any	
None	1716 (62)
1-5 d per 2 weeks	764 (28)
≥6 d per 2 weeks	280 (10)
VMS trajectory class, any	
Consistently low	704 (26)
Consistently high	846 (31)
Early onset	694 (25)
Late onset	512 (19)
Unknown	5 (0.2)
Race and ethnicity	
Black	737 (27)
Chinese	234 (8.5)
Hispanic	180 (6.5)
Japanese	265 (9.6)
White	1345 (49)
Education	
High school or less	601 (22)
Some college	871 (32)
≥4-y College	1265 (46)
Annual household income, US $	
Less than 10 000	139 (5.2)
10 000-19 999	195 (7.3)
20 000-34 999	412 (15)
35 000-49 999	490 (18)
50 000-74 999	639 (24)
75 000-99 999	376 (14)
100 000-149 999	310 (12)
≥150 000	126 (4.7)
Menopausal transition stage	
Premenopause	1524 (55)
Early perimenopause	1237 (45)
Body mass index, mean (SD)^a^	28 (7)
Smoking status	
Never smoked	1611 (58)
Past only	708 (26)
Current smoker	442 (16)
Alcohol use	
None	1323 (48)
<1 drink per week	302 (11)
1-7 drinks per week	728 (26)
>7 drinks per week	405 (15)
Physical activity score, mean (SD)^b^	7.73 (1.77)
Sex hormone-binding globulin, median (IQR), µg/mL	3.99 (2.76-5.51)
Testosterone, median (IQR), ng/dL	41 (30-56)
Free Androgen Index, median (IQR)	3.4 (2.1-5.8)

SI conversion factor: To convert testosterone to nmol/L, multiply by 0.0347; sex hormone-binding globulin to nmol/L, multiply by 8.896.

^a^ Calculated as weight in kilograms divided by height in meters squared.

^b^ Physical activity score is the sum of 3 indices of sports and exercise, active living, and household/caregiving domains of physical activity. The values of the score range from 3 to 15.

More frequent time-varying VMS were associated with increased risk of incident diabetes (relative to no VMS: frequent VMS HR, 1.45; 95% CI, 1.11-1.95; infrequent VMS HR, 1.30; 95% CI, 1.00-1.70), adjusted for covariates (Table 2). Four VMS trajectories were identified, with some patient falling into a fifth, unknown category: (1) consistently low probability of VMS (704 patients [26%]), (2) persistently high probability of VMS (846 patients [31%]), (3) early onset-initial high probability of VMS that decreased over time (694 patients [25%]), (4) late onset-initial low probability of VMS that increased over time (512 patients [19%]), and unknown (5 patients [0.2%]). Women with persistently high VMS had an increased risk of diabetes compared with consistently low VMS (HR, 1.50; 95% CI, 1.12-2.02). Results were similar for both hot flashes and night sweats (Table 2).

**Table 2.** Crude and Adjusted Hazard Ratio (HR) of Incident Diabetes by Frequency of Any Vasomotor Symptoms (VMS) at Baseline and Time-Varying and by VMS Trajectories^a^^,^^b^

Frequency or trajectory of symptoms	HR (95% CI)		
	Unadjusted	Adjusted model 1^b^	Adjusted model 2^c^
Frequency of any VMS			
Baseline: None	1 [Reference]	1 [Reference]	1 [Reference]
Baseline: 1-5 d per 2 weeks	1.17 (0.91-1.49)	1.07 (0.83-1.38)	1.03 (0.80-1.33)
Baseline: ≥6 d per 2 weeks	1.71 (1.26-2.35)	1.25 (0.91-1.73)	1.21 (0.88-1.67)
Time-varying: none	1 [Reference]	1 [Reference]	1 [Reference]
Time-varying: 1-5 d per 2 weeks	1.20 (0.93-1.56)	1.30 (1.00-1.70)	1.26 (0.97-1.63)
Time-varying: ≥6 d per 2 weeks	1.34 (1.02-1.77)	1.45 (1.11-1.95)	1.41 (1.05-1.87)
Frequency of hot flashes			
Baseline: none	1 [Reference]	1 [Reference]	1 [Reference]
Baseline: 1-5 d per 2 weeks	1.54 (1.20-1.98)	1.38 (1.06-1.78)	1.34 (1.04-1.73)
Baseline: ≥6 d per 2 weeks	1.61 (1.13-2.29)	1.20 (0.83-1.74)	1.20 (0.83-1.73)
Time-varying: none	1 [Reference]	1 [Reference]	1 [Reference]
Time-varying: 1-5 d per 2 weeks	1.17 (0.91-1.52)	1.27 (0.98-1.65)	1.23 (0.95-1.60)
Time-varying: ≥6 d per 2 weeks	1.27 (0.96-1.68)	1.35 (1.01-1.79)	1.30 (0.97-1.73)
Frequency of night sweats			
Baseline: none	1 [Reference]	1 [Reference]	1 [Reference]
Baseline: 1-5 d per 2 weeks	1.03 (0.79-1.34)	0.90 (0.68-1.18)	0.90 (0.68-1.18)
Baseline: ≥6 d per 2 weeks	1.56 (1.07-2.28)	1.14 (0.77-1.68)	1.09 (0.74-1.62)
Time-varying: none	1 [Reference]	1 [Reference]	1 [Reference]
Time-varying: 1-5 d per 2 weeks	1.09 (0.84-1.42)	1.18 (0.90-1.55)	1.14 (0.87-1.49)
Time-varying: ≥6 d per 2 weeks	1.44 (1.06-1.95)	1.55 (1.13-2.12)	1.46 (1.07-2.00)
VMS trajectories			
Any VMS			
Persistently high (n = 846)	1.47 (1.11-1.95)	1.50 (1.12-2.02)	1.42 (1.06-1.92)
Early onset (n = 694)	0.87 (0.62-1.20)	0.83 (0.59-1.16)	0.77 (0.56-1.08)
Late onset (n = 512)	0.76 (0.52-1.10)	0.98 (0.67-1.44)	0.97 (0.66-1.43)
Consistently low (n = 704)	1 [Reference]	1 [Reference]	1 [Reference]
Hot flashes			
Persistently high (n = 707)	1.51 (1.16-1.96)	1.64 (1.25-2.17)	1.56 (1.18-2.05)
Early onset (n = 658)	0.85 (0.62-1.15)	1.27 (0.92-1.75)	1.28 (0.93-1.76)
Late onset (n = 532)	1.03 (0.75-1.41)	1.02 (0.74-1.42)	0.94 (0.67-1.30)
Consistently low (n = 864)	1 [Reference]	1 [Reference]	1 [Reference]
Night sweats			
Persistently high (n = 514)	1.93 (1.36-2.73)	1.86 (1.30-2.67)	1.73 (1.21-2.48)
Early onset (n = 521)	1.49 (1.03-2.17)	1.33 (0.91-1.95)	1.22 (0.83-1.79)
Late onset (n = 1157)	1.43 (1.02-2.00)	1.21 (0.86-1.72)	1.23 (0.87-1.74)
Consistently low (n = 564)	1 [Reference]	1 [Reference]	1 [Reference]

^a^ Crude/unadjusted model includes site and site visit number as covariates.

^b^ Model adjusted for site, visit number, race and ethnicity, baseline age and education, time-varying body mass index, physical activity score, smoking status, alcohol consumption, and menopause transition stage.

^c^ Model further adjusted for sex hormone binding globulin and testosterone levels at baseline.

## Discussion

The study was limited to women with at least 3 visits before diabetes diagnosis to quantify trajectories; therefore, women who have a short time from VMS onset to diabetes are not represented. Women who had different patterns of VMS in the past 2 weeks from the other parts of the year may have been misclassified. SWAN did not measure hemoglobin A_1C_, which is typically used to diagnose diabetes.

In this prospective cohort study, frequent VMS or a trajectory of persistent VMS were associated with a 50% increased risk of diabetes. Women with frequent and/or persistent VMS over the MT may represent a high-risk group to target for diabetes prevention.

## Notes

Supplement 1. **eMethods. **Diabetes Ascertainment **eTable.** Number of SWAN Women Contributing to the Analysis at Each SWAN Visit
eReferences

Supplement 2.
Data Sharing Statement

## References

[1] Gray KE, Katon JG, LeBlanc ES, . Vasomotor symptom characteristics: are they risk factors for incident diabetes? Menopause. 2018;25(5):520-530. doi:10.1097/GME.0000000000001033 29206771 PMC5898980

[2] Gold EB, Colvin A, Avis N, . Longitudinal analysis of the association between vasomotor symptoms and race/ethnicity across the menopausal transition: study of women’s health across the nation. Am J Public Health. 2006;96(7):1226-1235. doi:10.2105/AJPH.2005.066936 16735636 PMC1483882

[3] Tepper PG, Brooks MM, Randolph JF Jr, . Characterizing the trajectories of vasomotor symptoms across the menopausal transition. Menopause. 2016;23(10):1067-1074. doi:10.1097/GME.0000000000000676 27404029 PMC5028150

[4] Sowers M, Crawford SL, Sternfeld B, . Chapter 11 - SWAN: A Multicenter, Multiethnic, Community-Based Cohort Study of Women and the Menopausal Transition. In: Lobo RA, Kelsey J, Marcus R, eds. Menopause. Academic Press; 2000:175-188. doi:10.1016/B978-012453790-3/50012-3

[5] Allison PD. Discrete-time methods for the analysis of event histories. Sociol Methodol. 1982;13:61-98. doi:10.2307/270718

[6] Jones BL, Nagin DS, Roeder K. A SAS procedure based on mixture models for estimating developmental trajectories. Sociol Methods Res. 2011;29(3):374-393. doi:10.1177/0049124101029003005

