# Supplementary material for: Vasomotor Symptom Trajectories and Risk of Incident Diabetes
Source: JAMA Netw Open. 2024 Oct 31;7(10):e2443546. doi: 10.1001/jamanetworkopen.2024.43546 (PMC11528338; doi:10.1001/jamanetworkopen.2024.43546)
Supplement: Supplement 1. — eMethods. Diabetes Ascertainment eTable. Number of SWAN Women Contributing to the Analysis at Each SWAN Visit eReferences [file jamanetwopen-e2443546-s001.pdf]

Supplemental Online Content

Hedderson MM, Liu EF, Lee C, et al. Vasomotor symptom trajectories and risk of incident diabetes. *JAMA Netw Open*. 2024;7(10):e2443546. doi:10.1001/jamanetworkopen.2024.43546

eMethods. Diabetes Ascertainment
eTable. Number of SWAN Women Contributing to the Analysis at Each SWAN Visit
eReferences

This supplemental material has been provided by the authors to give readers additional information about their work.

© 2024 Hedderson MM et al. *JAMA Network Open.*

eMethods. Diabetes Ascertainment

Serum glucose levels were measured at all clinic visits, except follow-up visits 9 and 10, using a hexokinase-coupled reaction (Boehringer Mannheim Diagnostics, Indianapolis, IN, USA). Use of anti-diabetic medication was determined by self-report and corroborated with visual inspection of medications at the study visit. Women were defined as having diabetes if they reported use of anti-diabetic medication at any time point during the study, had two consecutive visits with fasting glucose ≥126 mg/dL while not on steroids, or had any two visits with self-reported diabetes and a visit with fasting glucose ≥126 mg/dL. The SWAN visit at which diabetes was first observed/detected (visit for incident diabetes) was defined as: among women who used anti-diabetic medication, the first visit with serum glucose ≥126 mg/dL before first use of anti-diabetic medication; otherwise, the first visit with self-reported diabetes before first use of anti-diabetic medication; otherwise, the first visit at which the participant reported use of anti-diabetic medication. Among women who did not use anti-diabetic medication, the visit at which diabetes was first observed was defined as the first visit with fasting serum glucose ≥126 mg/dL while not on steroids. For participants [41(8%)] who had more than two missing visits with no fasting glucose or self-reported diabetes data between the first visit at which they used antidiabetic medication or had high fasting glucose ≥126 mg/dL and the assigned visit as described above, the first diabetes visit was imputed as the mean visit between these two visits. Types of diabetes (i.e., type 1 or type 2) were not determined, but most of the cases of diabetes in this life stage can be assumed to be type 2.

Covariate Definitions.

Standardized questionnaires were used to collect information on participants' age, race/ethnicity,[1] educational attainment[2], health behaviors (smoking status[2]), and MT status[3] and menopause hormone use[1,3-6]. Menopausal transition stage was categorized as: Premenopausal, perimenopausal or postmenopausal or unknown due to hormone therapy use or hysterectomy

© 2024 Hedderson MM et al. *JAMA Network Open.*

and included as a time-varying covariate. Physical activity was assessed using an adaptation of the Kaiser Physical Activity Survey.[7,8] Body mass index (BMI, kg/m^2) was calculated using height, measured by stadiometer, and weight, measured using a calibrated scale. BMI was categorized using standard adult BMI cut points for Black and White women[9] and using Asian-specific BMI cut points for Chinese and Japanese women:[10]

Detailed Statistical Analysis

Incident diabetes was modeled using a discrete-time survival framework anchored at the baseline visit because data were collected approximately annually. Participants who did not receive a diabetes diagnosis during the study period were considered censored at their last observed visit. The relationship between reported VMS frequency and incident diabetes was assessed over time as a time-varying exposure using separate discrete time hazard models[11] via fitted generalized linear models with a complementary log-log link to generate hazard ratios (HRs) and their 95% confidence intervals (CIs). The models were adjusted for time (visit number) to specify the baseline hazard function, and the following potential confounders chosen *a priori* based on scientific judgment: study site, race/ethnicity, baseline age, educational attainment, and time-varying covariates of menopausal transition stage, BMI, physical activity score, smoking status, and alcohol consumption. Only time-varying exposures and covariates collected before or at the visit at which first report of incident diabetes was captured (or last observed visit) were used in the analyses (see supplemental Table 1). Interactions between VMS frequency and race/ethnicity, menopausal transition stage and BMI over time were separately examined as cross-product terms.

© 2024 Hedderson MM et al. *JAMA Network Open.*

Time-varying VMS were also modeled using a group-based trajectory approach[12,13] to identify empirically clusters of participants with similar VMS patterns over the course of the study. Prior analyses in the full SWAN cohort identified four distinct trajectories.[14] For the present study, VMS trajectories were re-identified among our analytic sample among women with three or more visits with VMS data. Only VMS data prior to incident diabetes were used. Separate trajectory analyses were conducted for any VMS, night sweats only, and hot flashes only. Trajectory models assumed that VMS outcomes could be modeled by a polynomial function of time and were adjusted for study site and baseline age. Models with 1 to 7 groups, as well as quadratic and cubic polynomial trajectories were considered; selection of the final model was informed by the Akaike Information Criteria as a goodness-of-fit measure and alignment with previous studies using the SWAN cohort [14].

After implementing the trajectory modeling procedure, we plotted the final fitted trajectories (probability of time-varying VMS as a function of visit number). All three time-varying VMS measures (any VMS, night sweats only, hot flashes only) identified four trajectory classes. We then assigned the names of the trajectory classes based on the shapes of the plotted trajectories. To be more concrete, for the time-varying VMS measure of “any VMS,” one trajectory class had a probability of any VMS close to 1 during the entire follow-up period. Accordingly, we described this trajectory class as “persistently high probability of VMS” – an individual in this trajectory class likely had VMS symptoms during the entirety of the study period. In contrast, another trajectory class had a probability of any VMS close to 0 for the duration of follow-up: we described this trajectory class as “consistently low probability of VMS” – an individual in this

© 2024 Hedderson MM et al. *JAMA Network Open.*

trajectory class likely had no VMS symptoms over the course of the study period. A third trajectory class had a high probability (>0.75) of any VMS during the first half of follow-up that dropped monotonically to below 0.25 during the latter half of follow-up, which we described as “early onset-initial high probability of VMS that decreased over time.” The final trajectory class had a probability of any VMS near 0 at baseline that increased and peaked after 10-years of follow-up, which we described as “late onset-initial low probability of VMS that increased over time.” Participants were assigned a class based on their highest posterior probability.

A discrete time hazard model, again using a fitted generalized linear model with a complementary log-log link, estimated the relationship between VMS trajectory class and incident diabetes. The models were adjusted for time (visit number), study site, race/ethnicity, baseline age, baseline educational attainment, and time-varying covariates of menopausal transition stage, BMI, physical activity score, smoking status, and alcohol consumption. Interaction between VMS trajectory class and menopausal transition stage over time were examined as cross-product terms.

Sensitivity analyses were conducted. First, we excluded data from the New Jersey site to assess the impact of the site’s hiatus in data collection. Second, trajectories for any VMS were modeled by participant age and adjusted for study site. Four trajectory classes were identified with close alignment to the primary analysis and used to estimate the relationship with incident diabetes.

Analyses were conducted in RStudio. Specifically, the group-based trajectory models[12] were implemented using the LCMM package [15]. All results were considered statistically significant at a p-value ≤ 0.05.

**eTable. Number of SWAN Women Contributing to the Analysis at Each SWAN Visit**

© 2024 Hedderson MM et al. *JAMA Network Open.*

Visit Number														
**1**	**2**	**3**	**4**	**5**	**6**	**7**	**8**	**9**	**10**	**11**	**12**	**13**	**14**	**15**
2761	2761	2761	2694	2615	2528	2455	2388	2350	2265	2212	2156	2045	1971	1739

© 2024 Hedderson MM et al. *JAMA Network Open.*

eReferences

1. Centers for Disease Control and Prevention NCfHSVaHS. *Plan and Operation of the Third National Health and Nutrition Examination Survey, 1988-94*. 1994. DHHS Publication No. (PHS) 94-1308.
2. DHHS Publication No. (PHS) 94-1308 Plan and Operation of the Third National Health and Nutrition Examination Survey, 1988-94 (United States Department of Health and Human Services) (1994).
3. Gold E, Eskenazi B, Lasley B, al e. Epidemiologic methods for prospective assessment of menstrual cycle and reproductive characteristics in female semiconductor workers. *American Journal of Industrial Medicine*. 1995;28(6):783-797.
4. Ferris B. Epidemiology Standardization Project (American Thoracic Society). *American Review of Respiratory Disease*. 1978;118:1-120.
5. Matthews KA, Meilahn E, Kuller LH, Kelsey SF, Caggiula AW, Wing RR. Menopause and risk factors for coronary heart disease. *N Engl J Med*. Sep 7 1989;321(10):641-6. doi:10.1056/nejm198909073211004
6. Kuller LH, Matthews KA, Sutton-Tyrrell K, Edmundowicz D, Bunker CH. Coronary and aortic calcification among women 8 years after menopause and their premenopausal risk factors : the healthy women study. *Arteriosclerosis, thrombosis, and vascular biology*. Sep 1999;19(9):2189-98.
7. Sternfeld B, Ainsworth BE, Quesenberry CP. Physical activity patterns in a diverse population of women. *Prev Med*. Mar 1999;28(3):313-23. doi:10.1006/pmed.1998.0470
8. Baecke JA, Burema J, Frijters JE. A short questionnaire for the measurement of habitual physical activity in epidemiological studies. *Am J Clin Nutr*. Nov 1982;36(5):936-42. doi:10.1093/ajcn/36.5.936
9. Prevention CfDCa. About Adult BMI: How is BMI calculated and interpreted. Center for Disease Control and Prevention. Accessed October 3, 2014, http://www.cdc.gov/healthyweight/assessing/bmi/adult_bmi/index.html#Interpreted
10. Consultation WE. Appropriate body-mass index for Asian populations and its implications for policy and intervention strategies. *Lancet*. 2004;363(9403):157-163.
11. Allison PD. Discrete-Time Methods for the Analysis of Event Histories. *Sociological Methodology*. 1982;13:61.
12. Nagin DS, Odgers CL. Group-based trajectory modeling in clinical research. *Annu Rev Clin Psychol*. 2010;6:109-38. doi:10.1146/annurev.clinpsy.121208.131413
13. Jones BL, Nagin DS, Roeder K. A SAS procedure based on mixture models for estimating developmental trajectories. *Sociological Methods and Research*. 2011;29(3):374-393. NOT IN FILE.
14. Tepper PG, Brooks MM, Randolph JF, Jr., et al. Characterizing the trajectories of vasomotor symptoms across the menopausal transition. *Menopause*. Oct 2016;23(10):1067-74. doi:10.1097/GME.0000000000000676
15. Proust-Lima C, Philipps V, Liquet B. Estimation of Extended Mixed Models Using Latent Classes and Latent Processes: The R Package lcmm. *Journal of Statistical Software*. 06/01 2017;78(2):1 - 56. doi:10.18637/jss.v078.i02

© 2024 Hedderson MM et al. *JAMA Network Open.*
