# Supplementary material for: Vasomotor Symptom Trajectories and Risk of Incident Diabetes
Source: JAMA Netw Open. 2024 Oct 31;7(10):e2443546. doi: 10.1001/jamanetworkopen.2024.43546 (PMC11528338; doi:10.1001/jamanetworkopen.2024.43546)
Supplement: Supplement 2. — Data Sharing Statement [file jamanetwopen-e2443546-s002.pdf]

Data Sharing Statement

Hedderson. Vasomotor Symptom Trajectories and Risk of Incident Diabetes. *JAMA Netw Open*. Published October 31, 2024. doi:10.1001/jamanetworkopen.2024.43546

Data
Data available: No

Additional Information
Explanation for why data not available: It is not allowed by our IRB.
